# Anti-Apoptotic Machinery Protects the Necrotrophic Fungus *Botrytis cinerea* from Host-Induced Apoptotic-Like Cell Death during Plant Infection

**DOI:** 10.1371/journal.ppat.1002185

**Published:** 2011-08-18

**Authors:** Neta Shlezinger, Anna Minz, Yonatan Gur, Ido Hatam, Yasin F. Dagdas, Nicholas J. Talbot, Amir Sharon

**Affiliations:** 1 Department of Molecular Biology and Ecology of Plants, Tel Aviv University, Tel Aviv, Israel; 2 School of Biosciences, University of Exeter, Exeter, United Kingdom; Virginia Polytechnic Institute and State University, United States of America

## Abstract

Necrotrophic fungi are unable to occupy living plant cells. How such pathogens survive first contact with living host tissue and initiate infection is therefore unclear. Here, we show that the necrotrophic grey mold fungus *Botrytis cinerea* undergoes massive apoptotic-like programmed cell death (PCD) following germination on the host plant. Manipulation of an anti-apoptotic gene *BcBIR1* modified fungal response to PCD-inducing conditions. As a consequence, strains with reduced sensitivity to PCD were hyper virulent, while strains in which PCD was over-stimulated showed reduced pathogenicity. Similarly, reduced levels of PCD in the fungus were recorded following infection of *Arabidopsis* mutants that show enhanced susceptibility to *B. cinerea*. When considered together, these results suggest that *Botrytis* PCD machinery is targeted by plant defense molecules, and that the fungal anti-apoptotic machinery is essential for overcoming this host-induced PCD and hence, for establishment of infection. As such, fungal PCD machinery represents a novel target for fungicides and antifungal drugs.

## Author Summary

Our work provides a fundamental new insight into the biology and infection strategy of *Botrytis cinerea,* an economically important plant pathogen, which is used as a model for studying pathogenicity of necrotrophic plant pathogens. A fundamental question that we sought to address in this study is how *B. cinerea,* and possibly other necrotrophic pathogens, overcome the plant defenses in living host cells. For the first time we show evidence that help explain the basic events behind early stages of necrotrophic interaction. Our results show that plant defense molecules target the fungal PCD machinery and trigger massive cell death in the pathogen. Conversely, the fungal anti-apoptotic machinery, mediated by the IAP-like protein BcBir1, protects the fungus from host-induced PCD, thereby allowing establishment of small infection zones, from which spreading lesions will be produced at the second infection stage. Weakening or strengthening of *B. cinerea* anti-apoptotic machinery results in reduced or enhanced disease symptoms, respectively, whereas weakening of the plant defense system is associated with reduced fungal PCD and enhanced disease. While the current results are restricted to *B. cinerea,* we believe that our findings have important implications for understanding of fungal-host interactions, including the interplay between pathogenic fungi and animal hosts, particularly human pathogens.

## Introduction

Plant pathogenic fungi have evolved two prominent infection strategies: biotrophic pathogens proliferate within the living plant tissue deriving nutrition from living plant cells, and necrotrophic pathogens that do not occupy living plant cells and instead, kill host cells before tissue colonization. In order to infect plants, biotrophic pathogens suppress plant defenses [1], a strategy that is commonly used by pathogens to overcome host immunity [2], [3]. In contrast, necrotrophic pathogens secrete enzymes and toxins that kill the host tissue ahead of pathogen invasion, thus avoiding direct contact with defense molecules in living plant cells. It is unclear how this group of pathogens overcomes the host defenses during the early stages of infection, when the fungus is in contact with living host cells.

The grey mold fungus *Botrytis cinerea* has become an important model for the study of interactions between plants and necrotrophic pathogens [4]. *B. cinerea* infects over 200 cultivated plant species and causes significant economic damage to crops worldwide. Host-specific resistance is ineffective against *B. cinerea* and, conversely, the fungus utilizes the hypersensitive resistance response (HR) and the associated programmed cell death of the host to advance infection [5], [6]. The plant defense responses activated by *B. cinerea* are regulated in several ways, including the jasmonate and ethylene signaling pathways, as well as by additional signaling cascades that have not yet been identified [7], [8], [9]. Collectively, these defense responses can slow *B. cinerea* infection, but they do not completely block disease development [10], [11], [12].

Production of anti-microbial secondary metabolites represents an important component of plant defense responses against pathogens [13], [14]. In case of *B. cinerea,* the most significant secondary metabolites are glucosinolate, indolic, and phenylpropanoid compounds [15], [16]. Specifically, the indolic phytoalexin camalexin has been shown to play important role in defense of *A. thaliana* against a range of pathogens, including *B. cinerea* [10], [11], [15]. Production of camalexin is induced in plants following infection with *B. cinerea*, and *A. thaliana* mutant plants affected in camalexin biosynthesis show variable levels of enhanced sensitivity to pathogens [17]. The final step of camalexin production is catalyzed by a cytochrome P-450 monooxygenase encoded by the locus *PAD3* [18]; *pad3* mutant plants do not produce camalexin and are hypersensitive to a wide range of pathogens including *B. cinerea.* It has been proposed that *PAD3* is required for basal resistance to *B. cinerea* strains sensitive to camalexin [7], [15]. Although it has been shown that camalexin is toxic to a wide range of fungi, the mode of action remained unclear.

At least some plant antimicrobial compounds have the potential to induce apoptotic-like programmed cell death (PCD) in fungi [19], [20], [21]. Because necrotrophic fungi are unable to evade the plant defenses, we reasoned that plant defense molecules might target the fungal PCD machinery as a way to induce cell death in necrotrophic pathogens. Here we show that *B. cinerea* is exposed to massive, plant-induced PCD during the early phase of infection. Mutant plants that are affected in defense responses and are hypersensitive to *B. cinerea,* induce reduced levels of PCD in the fungus. We also show that camalexin induces PCD in *B. cinerea* and that it is important for the full scale host-driven PCD during the early stages of infection. The fungal anti PCD machinery attenuates camalexin and host-driven PCD and is therefore necessary for infection.

## Results

### Generation of apoptosis-attenuated fungal strains

To investigate the importance of PCD in *Botrytis* pathogenesis, we first generated *B. cinerea* strains showing both enhanced and impaired levels of PCD. In order to generate strains showing altered PCD, we isolated *BcBIR1* (BC1G_14521.1), a *B. cinerea* homolog of the *Saccharomyces cerevisiae BIR1* gene (GenBank P47134) (Figure S1). Bir1 is a component of the chromosomal passenger complex, which regulates chromosome segregation, an activity that is mediated by the carboxy-terminal domain of the protein and essential for yeast survival [22]. Bir1p also has anti-apoptotic activity, which is mediated by the two BIR domains at the N' terminal part of the protein and is not necessary for the cell cycle regulating activities of the protein [23]. The *B. cinerea* BcBir1 protein also contains two BIR domains at the N' part of the protein, it shares 24% of amino acid sequence identity with Bir1, but it is considerably shorter than Bir1 (601 amino acids and 954 amino acids, respectively).

Transgenic strains were first produced showing high level expression of the entire *BcBIR1* gene or an N' terminal region of the protein containing the two BIR domains (Figure S2). Complete deletion of *BcBIR1* was not possible because the gene is essential, similar to the *S. cerevisiae BIR1* gene. We therefore used a partial knockout (heterokaryon) strain (Δ*bcbir1*) in which the expression of *BcBIR1* was reduced, but not completely eliminated.

### PCD in *BcBIR1* transgenic strains

**Figure 1:**
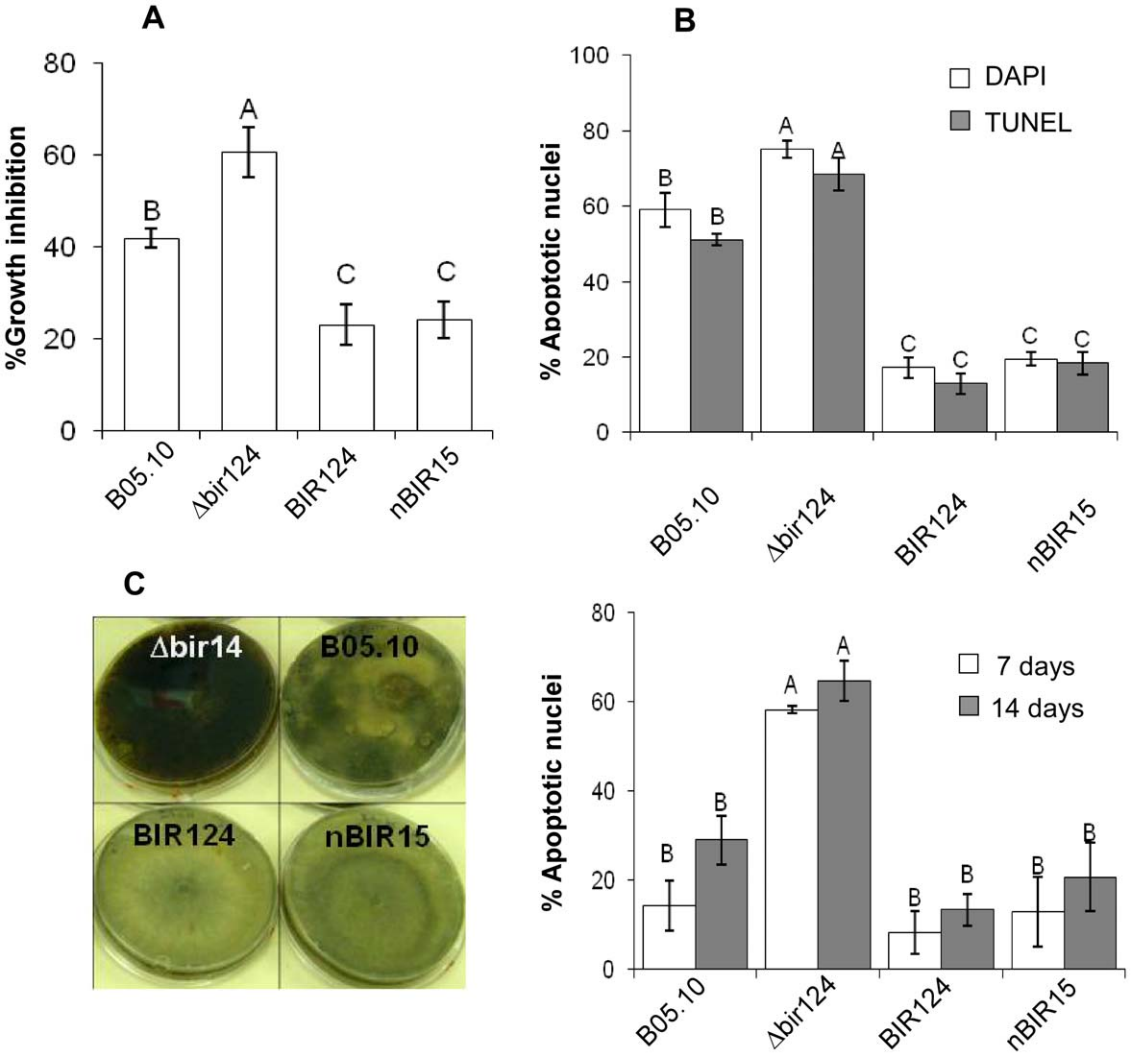
*BcBIR1* encodes a protein with anti-apoptotic activity. (**A, B**) PCD induced by H_2_O_2_ (see also Figure S3 for additional conditions). (**A**) Fungi were grown in PDB for 24 h, 8 mM H_2_O_2_ was added and relative biomass was determined after 48 h by measuring the OD of the cultures. Percent of growth inhibition was calculated by dividing the OD of treated and untreated cultures. Data shown represent mean ±SEM for four independent experiments performed in triplicates. (**B**) Accumulation of apoptotic markers. Chromatin condensation (DAPI) and DNA strand breaks (TUNEL) were recorded 4 h after treatment with H_2_O_2_. Data shown represent mean ±SEM for triplicate samples of 200 nuclei per sample (n = 3). Percentage of apoptotic nuclei in untreated cultures were less than 0.5% for WT, BcBir124 and nBir15, and lower than 7% (DAPI) and 4% (TUNEL) for Δbir14. (**C**) Ageing. Cultures were maintained under normal growth conditions, spores were collected after 7 and 14 days, stained with DAPI, and percent of apoptotic nuclei was determined. Data shown represent mean ±SEM for triplicate samples of 100 spores per sample (n = 4). Columns and lines not connected with the same letter are statistically different (p<0.05) as determined by one-way ANOVA (p<0.001) followed by a post-hoc Tukey HSD analysis. Pictures show increased accumulation of black pigments, which indicates senescence of the cultures in the Δ*bcbir1* mutant. The strains were cultured on PDA plates as described above. Pictures were taken after 14 days of culturing.

Next, we tested the ability of these *B. cinerea* strains to undergo PCD. When treated with apoptosis-inducing agents such as H_2_O_2_, or under physiological conditions that induce PCD such as ageing or in stationary phase [19], [24]. *BcBIR1* over-expression strains (expressing either the entire protein or only the N' part) retained higher growth rates and showed reduced PCD markers (chromatin condensation, DNA strand breaks, ROS accumulation), whereas Δ*bcbir1* mutant strains exhibited reduced growth rates and showed enhanced PCD compared with the isogenic wild type strain (Figures 1, S3). These results show that BcBir1 has anti-apoptotic activity, which is mediated by the BIR domains at the N' terminal part of the protein, similar to the *S. cerevisiae* homologue Bir1p.

### The *BcBIR1* mutant strains are affected in pathogenicity

**Figure 2:**
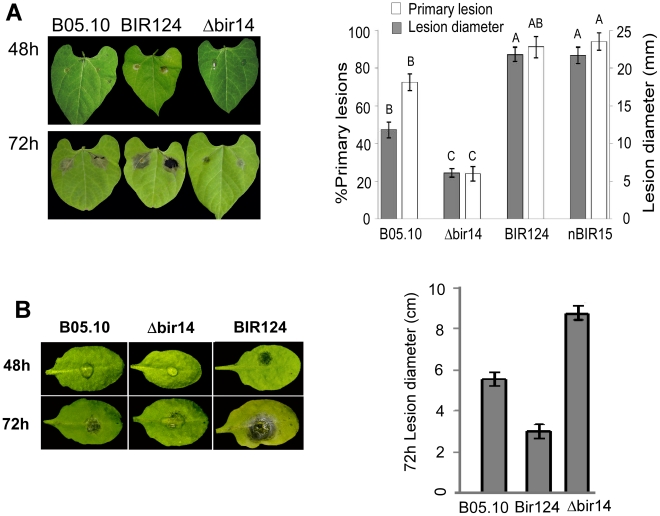
*BcBIR1* mutant strains are affected in pathogenicity. Leaves of *P. vulgaris* (**A**) and *A. thaliana* (**B**) were inoculated with *B. cinerea* B05.10 wild type strain, Δ*bir14* mutant strain and BIR124 and nBIR15 strains that over-express the entire *BcBIR1* or the N' part of the protein, respectively. Percent of primary lesions on beans (*P. vulgaris*) leaves was recorded 48 h PI by counting the number of droplets that produced lesions. Size of expending lesions (on beans and Arabidopsis) was recorded 72 h PI. Data represent mean ±SEM of 32 droplets from four different plants per each fungal genotype (n = 4). Columns not connected with the same letter are statistically different (p<0.05) as determined by one-way ANOVA (p<0.001) followed by a post-hoc Tukey HSD analysis.

To test the effect of *BcBIR1* on pathogenicity, we inoculated beans leaves and measured disease symptoms. The *BcBIR1* over-expression strains (in which PCD was attenuated) caused increased disease symptoms, while Δ*bcbir1* mutant strains (in which PCD was enhanced) caused restricted lesions (Figure 2A). Similar results were observed on *Arabidopsis thaliana* (Figure 2B) and on a number of additional plant species that are host for *B. cinerea,* such as peas, tomato, tobacco and basil (data not shown). Thus, PCD and pathogenicity in *BcBIR1* transgenic strains are modified such that enhanced or reduced PCD is associated with reduced or enhanced pathogenicity, respectively. These results support the possibility that the fungus undergoes apoptotic-like PCD during infection, which might have an impact on disease establishment.

### Use of H1-GFP tagged strains to follow fungal infection

To test whether the fungus undergoes apoptotic-like PCD during infection, we generated a transgenic *B. cinerea* strain (H1-GFP), in which nuclei were tagged with GFP, and followed changes in the nuclear GFP signal in fungal hyphae during progression of plant infection. Cytoplasmic localization of the histone H1 was shown to play an important role during apoptosis in mammals [25], [26]. Furthermore, the disappearance of the nuclear signal in H1-GFP tagged strains has previously been used to indicate autophagic programmed cell death in *Magnaporthe oryzae* during appressorium formation [27], and apoptotic-like programmed cell death in *Colletotrichum gloeosporioides* following treatment with apoptosis-inducing agents [28].

First, we tested the effect of H_2_O_2_ on the GFP signal in the *B. cinerea* H1-GFP strain and compared it with accumulation of apoptotic markers, DNA strand breaks (determined by TUNEL) and chromatin condensation and fragmentation (determined by DAPI staining). During the first hours after treatment with H_2_O_2_, the nuclear GFP signal faded out and it could no longer be detected 12 h after treatment (Figure S4A). At this time point, as well as at 6 h post treatment, green fluorescence could be visualized in the entire hyphae, which might be attributed to dislocation of the GFP-tagged histone to cytoplasm during apoptosis, as was shown for mammalian cells [25], [26]. Alternatively, the green fluorescence in damaged hyphae at this stage might also result from disintegration of the nuclei, or due to auto-fluorescence of destroyed hyphae. DAPI staining showed enhanced staining of nuclei 6 and 12 h post H_2_O_2_ treatment, which is indicative of chromatin condensation (Figure S4A). Since it is impossible to distinguish between the TUNEL and GFP signals (in both cases nuclei fluoresce in green), we performed TUNEL assay on *B. cinerea* H1-GFP hyphae 12 h after treatment with H_2_O_2_, when the nuclear H1-GFP signal completely disappeared (Figure S4B). At this time point there was intense TUNEL staining, further demonstrating the correlation between loss of the nuclear GFP signal in the H1-GFP strain and progression of PCD. These analyses confirmed that loss of the nuclear GFP signal in the H1-GFP strain is correlated with early stages of apoptotic-like cell death.

**Figure 3:**
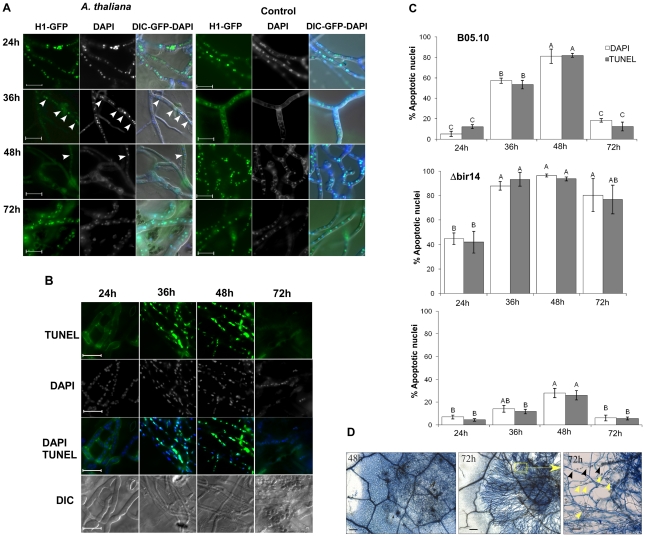
Disease symptoms are negatively correlated with PCD in *Botrytis*. (**A**) Plants were inoculated with the *B. cinerea* H1-GFP. Images show gradual decrease in the number of fluorescent nuclei between 24–48 h, and then recovery of the signal at 72 h. Very few nuclei are visible at 48 h (arrowhead). Control panels (right) show hyphae that were developed following spore germination on PDA medium. Abundant nuclei signal are observed at all times. (**B**) TUNEL and DAPI staining of *B. cinerea* on infected leave of *A. thaliana*. Leaves of Col-0 plants were inoculated with *B. cinerea* B05.10 wild type strain and TUNEL assay was performed to determine PCD in the developing hyphae. The green fluoresce of nuclei in these images indicate positive TUNEL staining of nuclei, *i.e.,* apoptotic cells. Bar  =  20 µm. (**C**) Levels of PCD during infection of *A. thaliana* Col-0 wild type plants with the *B. cinerea BcBIR1* mutant strains. Col-0 plants were inoculated with B05.10 wild type, Δ*bir14* mutant and BIR24 over expression strains. PCD was recorded by DNA strand break (TUNEL assay) and chromatin condensation (DAPI staining). Data represent mean ±SEM for triplicate samples of 200 nuclei per sample (n = 3). The effect of fungal genotype and infection phase on PCD level was determined using two-way ANOVA. Columns not connected with the same letter are statistically different (p<0.05) as determined by two-way ANOVA (p<0.001) followed by a post-hoc Tukey HSD analysis. (D) Typan blue staining of *Botrytis-*infected *Arabidopsis* leaves. Leaves of *A. thaliana* Col-0 plants were inoculated with a spore suspension of *B. cinerea* B05.10. Leaves were stained with trypan blue 48 h and 72 h PI. Blue staining of plant cells surrounding the fungal mycelium indicates dead plant cells. The right panel shows a higher magnification of the inset from the middle panel (both from 72 h PI). Degradation of the leaf veins (black arrowheads) and accumulation of vesicles that are produced from remnants of plant cells (yellow arrowheads) show complete maceration of the tissue in the center of the lesion. Bars in first two panels (48 h and 72 h)  =  100 µm, bar in last panel (inset 72 h)  =  20 µm.

We then used the H1-GFP strain to determine the fate of the fungal cells during plant infection. *A. thaliana* Col-0 plants were inoculated with conidia of the H1-GFP strain and GFP signal in fungal hyphae was monitored using live cell imaging during the first 72 h of plant infection (PI). The nuclear GFP signal was retained for the first 24 h PI, but during the next 24 h (24–48 h PI) it dissociated; some GFP fluorescence was first observed in entire hyphae (as was also seen following H_2_O_2_ treatment) and later faded out, consistent with progression of PCD within proliferating hyphae (Figure 3A). In accordance with results of the H_2_O_2_ treatment, significant reduction in the number of DAPI stained nuclei, which represents a final stage of cell death, lagged behind the disappearance of the H1-GFP signal and could only be observed at 48 h PI (Figure 3A, 48 h, Figure S4D).

Very few GFP-labeled nuclei could be detected at the 48 h time point in isolated fungal cells within the mass of mycelium in the necrotic zone (Figure 3A, 48 h arrowhead). However, despite massive cell death at 48 h, the fungus quickly recovered as indicated by reappearance of the nuclear signal at 72 h PI. Similar results were obtained on additional host plants, such as beans and peas (Figure S4C). This sequence of events only occurred on plants; when germinated on a glass slide, a stable nuclear signal was retained at all time points (Figure 3A, control). Thus, following germination and establishment of contact with the host, nuclear signal in H1-GFP strain starts to disappear between 24–36 h PI, and almost no nuclei can be detected by 48 h PI. Since disappearance of the H1-GFP signal is correlated with accumulation of apoptotic markers, these results suggest that the majority of fungal cells are exposed to plant-induced PCD during the early infection phase, when the fungus is in direct contact with living plant tissue.

We observed a similar pattern (though on a different time scale) of disappearance and reappearance of the H1-GFP nuclear signal during infection of corn plants by the necrotrophic fungus *Cochliobolus heterostrophus* (Figure S5, *Cc-Zm*). In contrast, in the hemibiotrophic pathogen *C. gloeosporioides* the H1-GFP signal was stable throughout the infection process on pea leaves (Figure S5, *Cg-Ps*). Significantly, inoculation of pea leaves with *B. cinerea* produced similar results to inoculation of beans (Figure S4C), indicating that the differences in H1-GFP signal did not result from different host responses and are therefore specific to the fungus. Further, similar to *C. gloeosporioides*, the nuclear signal was stable throughout infection also in *M. oryzae,* a hemibiotrophic fungus that infects rice and barley (Figure S5, *Mo-Os*). Thus, retention or loss of the H1-GFP signal is not a universal phenomenon of fungal pathogenesis, but rather it is specific to *B. cinerea*, and possibly to other necrotrophic pathogens.

### PCD is induced in Botrytis during the early infection phase

To verify that the results obtained with the H1-GFP strain are indicative of PCD, we measured DNA strand breaks (TUNEL positive nuclei) and chromatin condensation (condensed nuclei following DAPI staining) during infection of *A. thaliana* plants by the wild type B05.10 strain. In accordance with the results obtained with the H1-GFP marker, the level of PCD markers increased between 24 h and 48 h PI, and then reduced to near baseline levels at 72 h (Figure 3B–C). These results support the results obtained with the H1-GFP strain and confirm that massive PCD occurs during the early infection phase, but that the fungus manages to recover upon transition to the second phase.

### Modified PCD in *BcBIR1* mutant strains during plant infection

Infection of *A. thaliana* wild type plants with *BcBIR1* over-expression strains led to larger necrotic lesions whereas the Δ*bcbir1* mutant strain caused attenuated disease symptoms (Figure 2B). If PCD was a limiting factor in disease development, intensified and reduced disease symptoms are expected to be paralleled by reduced or increased levels of PCD. In support of this rational, the amount of PCD in the *BcBIR1* over-expression and Δ*bcbir1* mutant strains on infected Col-0 plants was reduced or enhanced, respectively (Figure 3C). These results further support the role of *BcBIR1*-mediated anti-apoptotic response as a mechanism to cope with plant-induced PCD during the direct contact of the fungus with living host cells.

### Attenuated fungal PCD on Botrytis-hypersensitive plants

**Figure 4:**
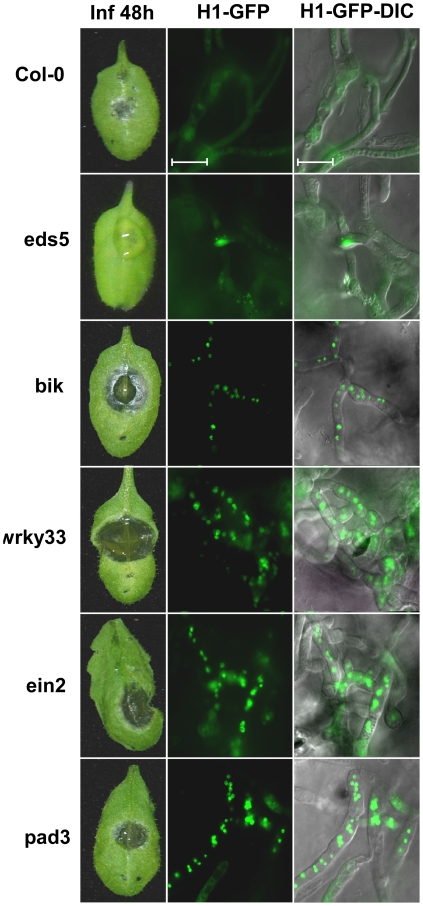
Lower PCD levels and enhanced pathogenicity of *B. cinerea* on *A. thaliana* immune-deficient plants. Leaves of all the plant lines were inoculated with spore suspension of the H1-GFP strain. Symptoms and GFP signals were recorded 48 h PI. Col-0 – wild type, *eds5* – a mutant in SA signaling with slightly reduced sensitivity to *B. cinerea* (both the Col-0 and eds5 are used a controls), *bik1, wrky33, ein2, pad3* are mutant lines with defects in the immune response (see text for details) and are hyper sensitive to *B. cinerea*. Enhanced pathogenicity on these mutants is paralleled by retention of intact nuclei (H1-GFP images).

To further investigate whether fungal PCD was induced by host defenses, we used a collection of *A. thaliana* mutant plants that represent mutations in a range of defense responses, all of which are hypersensitive to *B. cinerea*; *ein2* (ethylene signaling), *bik1* (receptor-mediated basal defense), *wrky33* (transcriptional regulation/MPK4-mediated signaling pathway) and *pad3* (camalexin production). When the *A. thaliana* mutant plants that are hyper-sensitive to *B. cinerea* were inoculated with the H1-GFP strain, the nuclear GFP signal was retained also at 48h, and enhanced disease symptoms developed (Figure 4), confirming that the amount of fungal PCD negatively correlates with plant susceptibility to the fungus.

### Induced fungal PCD by camalexin

**Figure 5:**
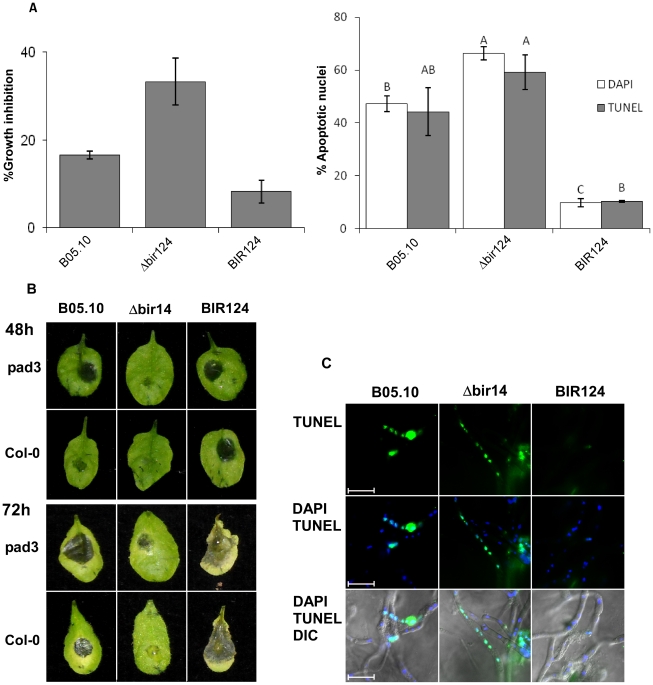
Camalexin induces PCD in *B. cinerea* and limits lesion spreading. (**A**) Growth inhibition and induction of PCD in *B. cinerea* by camalexin. Fungi were grown in PDB for 24 h and then camalexin was added at 15 µg/ml. **A**. Effects on growth (left graph) and PCD (right graph) were recorded after 48 h and 4 h, respectively. Growth assays were performed in triplicates. Apoptotic nuclei counts were determined by counting 200 nuclei per sample. Data represent mean ±SEM (n = 3). Columns not connected with the same letter are statistically different (p<0.05) as determined by one-way ANOVA (p<0.001) followed by a post-hoc Tukey HSD analysis. (**B**) Infection of camalexin-deficient *pad3* mutant and Col-0 wild type plants by *B. cinerea*. Plants were inoculated with spore suspensions of *B. cinerea* B05.10, Δ*bir14* mutant and BIR24 over expression strains. (**C**) PCD was detected by TUNEL assay 48 h PI. Note that in this case the green nuclear signal denotes apoptotic nuclei. Bar  =  20 µm.

*A. thaliana pad3* plants do not produce camalexin, the principal phytoalexin in *A. thaliana* [29]. Because *B. cinerea* undergoes reduced PCD on *pad3* mutant plants, it is possible that camalexin might be involved in the induction of fungal PCD. To determine whether camalexin induces PCD in *B. cinerea*, we treated mycelium with purified camalexin and measured growth and levels of PCD. Consistent with previous reports [10], 20% growth inhibition of the wild type strain was induced by exposure to camalexin at 15 µg/ml, which was accompanied by appearance of apoptotic markers (Figure 5A). The *BcBIR1* over expression and mutant strains showed reduced or enhanced sensitivity to camalexin respectively, along with reduced or enhanced PCD. We conclude that camalexin induces PCD in *B. cinerea.*

When inoculated with Δ*bcbir1* mutant strain, *pad3* plants developed clear necroses 72 h PI, unlike wild type Col-0 plants in which the initial lesions that were formed following inoculation with Δ*bcbir1* did not develop into spreading disease lesions (Figure 5B). Direct assays confirmed that in comparison to Col-0, on *pad3* mutant plants the wild type and Δ*bcbir1* fungal strains showed reduced levels of PCD, consistent with intensified disease symptoms (Figures 5C, S6) Notably, the *BcBIR1* over-expression strains showed little difference in plant infection and PCD on *pad3* plants compared with Col-0 plants, possibly due to the reduced sensitivity of these strains to camalexin-induced PCD and thus reduced PCD and enhanced pathogenicity on the wild type plants.

## Discussion

In this report we have shown that in the broad host range necrotrophic fungus *B. cinerea*, and possibly in additional necrotrophic fungi, massive PCD of fungal cells is induced by the plant defense response during the early phase of plant infection. An anti-apoptotic machinery is essential for protection of the fungus during this stage, and hence for establishment of initial infection.

A major element in the *B. cinerea* anti-apoptotic machinery is the protein BcBir1. Unlike most other fungal homologues of animal apoptotic genes that have been analyzed, which usually have only partial effect on PCD, our results show that BcBir1 plays major role in PCD, and that it is essential for function of the anti-apoptotic machinery. This allowed us to generate a set of transgenic strains with attenuated PCD through manipulation of *BcBIR1.* We used these strains together with a strain expressing the H1-GFP nuclear marker, to measure changes in fungal PCD during plant infection and determine the role of PCD, particularly the anti-apoptotic machinery of the fungus, in disease development.

**Figure 6:**
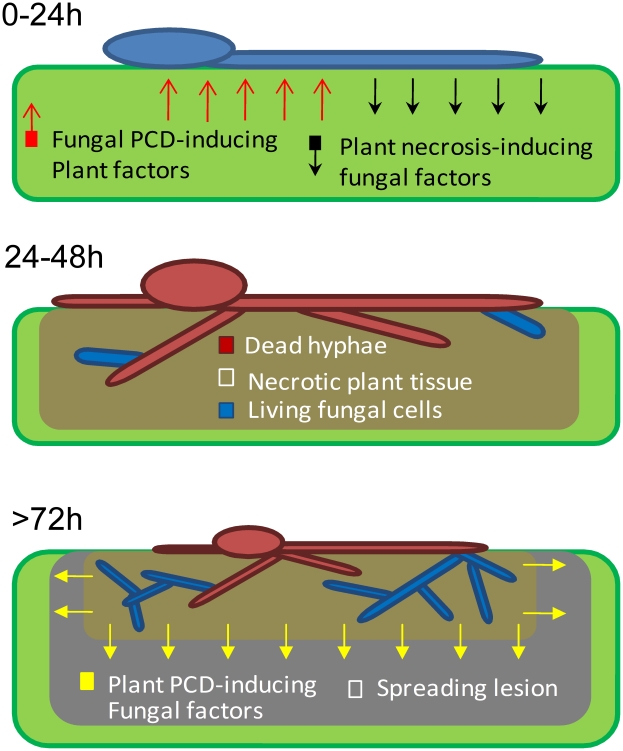
A model showing the role of PCD in early events of *B. cinerea* infection. 0–24: Following landing on the host, conidia germinate and start secreting necrosis-inducing factors. 24–48: Anti-fungal plant metabolites trigger PCD in developing hyphae, which kill most of the fungal cells. Anti-apoptotic machinery (mediated by BcBir1) prevents complete elimination of the fungus at this stage. >72: Viable fungal cells retained within the necrotized plant tissue are protected from the host toxic molecules. These cells give rise to new hyphae which secrete molecules that induce PCD in surrounding plant tissue and promote lesion spreading.

Rapid PCD of plant cells around the site of pathogen invasion is the main component of the HR resistance response, which is effective against specific biotrophic pathogens. In contrast, the HR response is rather ineffective against necrotrophic pathogens, which induce spreading cell death in infected plants, partly by manipulation of the plant's natural PCD response [1]. We have shown that in the broad host range fungus *B. cinerea*, rapid apoptotic-like PCD of fungal cells at the primary infection site is induced by the plant defense. The anti-apoptotic machinery in the fungus blocks host-induced PCD between 24–48 h PI, thereby preserving a small number of viable fungal cells within the necrotic zone. According to our model (Figure 6), surviving fungal cells within the destroyed plant tissue are sufficient to give rise to new mycelium, which initiates spreading lesions in the second phase of infection. Consistent with this model and in agreement with previous reports [11], staining of infected leaves with trypan blue (which stains dead cells) showed that fungal mycelium was restricted to areas of destroyed tissue, which were surrounded by a ring of dead plant cells that expanded as the lesion progressed (Figure 3D). Simulations on PDA plates show that a single viable cell is sufficient to give rise to a micro-colony composed of loosely packed, but elongated (1–2 cm) hyphae (not shown). Accordingly, fungal cells that remain viable at 48 h within the necrotic zone, and thus are protected from the host defense, are potentially capable of producing abundant hyphae by 72 h. Furthermore, the plant tissue within the necrotic region is massively degraded at 72 h (Figure 3D). Thus, inside the necrotic spot the fungus is protected from the host defense and has ample supply of nutrients, conditions which are supportive of intensive growth during the second infection phase. Spreading lesion is induced by fungal activity in the periphery, while development of new mycelium is restricted to areas of dead tissue (Figure 3D, 72 h, Figure 6).

A similar trend of H-1GFP signal disappearance and reappearance was observed in the corn necrotrophic pathogen *C. heterostrophus*, but not in two hemibiotrophic pathogens, *M. oryzae* and *C. gloeosporioides,* in which the nuclear signal was stable at all stages of infection. These results suggest that the phenomenon discovered in *B. cinerea,* namely, massive cell death followed by recovery of the fungus, might be a common strategy in necrotrophic pathogens; because pathogens in this class are unable to evade the host defense, and since host defense molecules target and trigger fungal PCD, the fungal anti-apoptotic machinery serves as a virulence mechanism by protecting the fungus from plant-induced PCD. Following the establishment of a protected infection zone, the fungus induces PCD in surrounding host tissues to promote disease spreading [4], [6], [30].

Plant and animal pathogens have evolved common features including conserved systems for deploying virulence proteins and convergent pathogenic strategies [2], [31], [32]. Increasing evidence suggest structural and functional similarities in microbial-host interaction mechanisms of plants, insects and vertebrates [3], [33], [34]. Particularly, a few studies have shown that PCD of eukaryotic pathogens of insect and mammals occur during host invasion [35], [36], [37]. Thus, anti-apoptotic activity of the pathogen might be important for host invasion in various other systems including human pathogens. Accordingly, metabolites that target the fungal PCD machinery might be valuable in developing novel antifungal drugs and in engineering plant resistance against economically significant fungal pathogens.

## Materials and Methods

### Fungal strains, growth conditions, and DNA analysis

*B. cinerea* wild type strain B05.10 and H1-GFP transgenic strain were used throughout this study. The H1-GFP strain carries the eGFP gene fused to the C-terminus of the histone E11-encoding gene hH1 from *Neurospora crassa* under control of *B. cinerea* actin promoter. The strain displays fluorescent nuclei and is hygromycin resistant. In addition we used transgenic strains of *Cochliobolus heterostrophus* c4 that express an hH1-eGFP gene fusion under control of *B. cinerea* actin promoter (this work), and *C. gloeosporioides* f. sp. *aeschynomene* strain 3.1.3 [28] and *M. oryzae* strain Guy11 [27] that express an hH1-RFP gene fusions, both under control of *N. crassa* CCG1 promoter. *B. cinerea* strains were routinely cultured on potato dextrose agar (PDA, Acumedia) and maintained at 21°C under fluorescent light. Conidia were obtained from 7–10 – days old cultures. Mycelium was produced from cultures that were grown in potato dextrose broth (PDB, Acumedia) in 250 ml Erlenmeyer-flasks, with agitation at 180 rpm. Growth assays were conducted in 24-wells plates.

Gel electrophoresis, restriction enzyme digestion, PCR and DNA gel blots hybridizations were all carried out using standard procedures [38]. DNA-mediated transformation of *B. cinerea* was carried out as previously described [39], [40]. DNA and RNA extraction, and cDNA synthesis were performed as previously described [41].

### Oligonucleotides and construction of plasmids DNA

The *B. cinerea* B05.10 genome sequence at BROAD (http://www.broad.mit.edu) was used to design primers for amplification of *BcBIR1* ORF and flanking sequences (Table S1). *BcBIR1* 5′ and 3′ flanking sequences (800 bp each) were amplified by PCR using primers 3/4 (5′) and 5/6 (3′). The two fragments were cloned into pUC57R/T (Fermentas), excised with *Not*I/*Sma*I (5′ flank) and *Mlu*I/*Sac*I (3′ flank), and cloned into pOliHP to produce pΔ*bcbir1* gene replacement vector. Over expression vectors were constructed with *BcBIR1* cDNA. A full length cDNA clone (1803 bp) was amplified by PCR using primers 1/2. *B. cinerea* ActA promoter (Bc_08198.1; 1,500 bp) and β-glucanase terminator (BC1T_00642; 500 bp) were amplified from genomic DNA using primers 14/15 and 11/12, respectively. The three PCR fragments were cloned into the corresponding restriction sites of pUC57R/T, the cassette was excised with *Hind*III and *Spe*I restriction enzymes and sub-cloned into pBluescript KS*+* to produce the KS-BcBIR1 plasmid. The Hygromycin resistance cassette from pOliHP vector was digested with *Not*I and *Sac*I and sub-cloned into KS-BcBIR1 to produce the *BcBIR1* over-expression vector pKSHAIG. For over-expression of the N' part, nucleotides 0 - 891 of *BcBIR1* containing both Bir domains were amplified with primers 1/13 and cloned into *Pac*I/*Asc*I sites in pKSHAIG in place of the *BcBIR1* ORF to produce the BcBIR1 N' over expression vector pKSHANIG.

### Plant infections

Infection assays of beans (*Phaseolus vulgaris* cv. French bean, genotype N9059) plants with *B. cinerea* were performed according to [39]. Infection levels were estimated by counting primary lesions 24–48 h PI (early phase) and measurement of lesions' diameter 72 or 96 h PI (late phase). Inoculation of *A. thaliana* plants with *B. cinerea* was performed in a similar way by placing a 6 µl droplet of spore suspension on each leaf. Wild type Columbia (Col-0) plants were routinely used. We also used the *A. thaliana* mutant plants *ein2* [42], *bik1* [12], *wrky33* [9], and *pad3* [17], all of which are affected in defense response genes that confer increased sensitivity to *B. cinerea*. In addition, the *eds-5* mutant, which has slightly reduced levels of sensitivity to *B. cinerea* was used as control. Pea (*Pisum sativum*, cv. white sugar) plants were cultivated and inoculated with *C. gloeosporioides* or *B.cinerea*, rice plants (*Oryza sativa* cv CO-39) were cultivated and inoculated with *M. oryzae*, and corn (*Zea maize* cv Jubilee) plants were cultivated and inoculated with *C. heterostrophus* as previously described [27], [43], [44]. Trypan blue staining was used to detect plant cell death following fungal infection [45]. Infected leaves were placed in lactophenol-trypan blue stain solution (1 mL of lactic acid, 1 mL of glycerol, 10 g of phenol, 10 mg of trypan blue, dissolved in 10 mL of distilled water) and boiled for 1 min. The stained leaves were decolorized in chloral hydrate (2.5 g of chloral hydrate dissolved in 1 mL of distilled water) for 30 min and then mounted on microscope slides and observed by bright filed microscope.

### Apoptosis assays and staining procedures

Apoptotic-like cell death was determined by measurement of accumulation of reactive oxygen species (ROS), chromatin condensation and amount of DNA-strand breaks, criteria that are commonly used to determine apoptotic PCD in fungi [24], [46]. All assays were performed in triplicates. Isolates were grown overnight before application of PCD-inducing compounds. Growth response was determined by measurement of the optical density using a synergy HT plate reader (Biotek). Intracellular ROS levels were detected by staining with Dihydro-rhodamine 123 (DHR123) (Sigma). Tissue was incubated for 3 h in PDB medium with 4 µg/ml DHR123 with agitation. Following incubation, samples were washed twice with ddH_2_O and then observed under fluorescent microscope using a rhodamine filter. Relative ROS levels were determined by measurement of fluorescence intensity divided by the relative biomass (both measurements were performed with a Biotek synergy HT plate reader). Chromatin condensation was detected following nuclear staining. Nuclei were stained with DAPI as previously described [43] or by incubation for 10 min with 15 µg/ml Hoechst 33342 (Sigma). Samples were visualized by fluorescent microscope using a DAPI filter. DNA strand breaks were detected by terminal deoxynucleotidyl transferase dUTP nick end labeling (TUNEL) using the *In Situ* Cell Death Detection kit (Roche Applied Science, Indianapolis, IN), as previously described [43]. Briefly, mycelia and infected leaves were fixed with 3.7% formaldehyde, digested with lysing enzyme (sigma), rinsed twice with PBS, incubated with 50 µl TUNEL reaction mixture for 70 min at 37°C with agitation and then rinsed 3 times with PBS. Samples were examined using a GFP filter. The percent of condensed and TUNEL positive nuclei was calculated by counting 200 cells/nuclei per sample.

### Microscopy

Epifluorescence and light microscopy were performed with a Zeiss Axio imager M1 microscope. Differential interference microscopy (DIC) was used for bright field images. The following filters were used for examination of fluorescent samples: DAPI filter (excitation 340–390 nm, emission 420–470 nm), rhodamine filter (excitation 540–552 nm, emission 575–640 nm), GFP filter (excitation 450–490 nm, emission 500–550 nm). Images were captured with a Zeiss AxioCam MRm camera and analyzed using Axiovision Rel 4.5 software package. Further processing and pixel intensity measurements were performed using the ImageJ 1.42q software (http//rsb.info.nih.gov/ij/).

### Statistical analysis

Statistical tests were applied using JMP 7.0.2 (SAS Institute, Cary, NC). Data were analyzed by analysis of variance (one-way or two-way ANOVA), followed by post hoc Tukey test for comparisons among genotypes. In all graphs, results represent the mean value of 3–5 independent experiments ± SEM. Columns marked with the same letters do not differ from each other at the significance level of *P*<0.05 (α = 5%) according Tukey HSD test for post-ANOVA analysis (p<0.001).

## Supporting Information

Figure S1 **The** ***B. cinerea BcBIR1*** **is a homologue of** ***BIR1*** **from** ***S. cerevisiae*** **.** (**A**) Alignment of the amino acid sequence of the two BIR domains in the predicted *B. cinerea* BcBir1 protein with BIR domains of *S. cerevisiae* (P47134) and *S. pombe* (CAA20434) Bir1p, and human XIAP (NP_001158), c-IAP (NP_001157) and survivin (NP_001159). The alignment was generating using ClustalW. Numbers on the top indicate amino acid residue positions. The conserved C2HC residues are marked by black squares. (**B**) A diagram comparing the organization of BIR domains in human XIAP (Type I IAP) and Survivin (Type II IAP), *S. cerevisiae* Bir1p and *B. cinerea* BcBIR1. The BIR domains are colored dark gray. The black square in XIAP denotes a RING domain.
(TIF)

Figure S2 **Generation of** ***BcBIR1*** **over expression and knockout strains.** (**A**) Diagrams of the *BcBIR1* knockout vector and genomic locus. Primers for PCR analyses and vector construction are marked by arrows. (**B**) RT-PCR analysis of hygromycin-resistant colonies obtained from transformation with the *BcBIR1* over expression vector pKSHAIG (BIR14, BIR124) and the *BcBIR1* N' part over expression vector pKSHANIG (nBIR13, nBIR15). OHGG denotes a transgenic strain expressing free GFP. (**C**) Southern blot analysis of hygromycin-resistant colonies obtained from transformation with the *BcBIR1* replacement vector pΔ*bcbir1*. Genomic DNA from hygromycin-resistant transformants and the wild type strain were digested with *Bcl*I. Blots were probed with the fragment that is marked by a dashed line in (**A**). Wild type nuclei produce a band of 3.9 kb, gene replacement events produce a band of 4.8 kb.
(TIF)

Figure S3 **Apoptotic markers in wild type and** ***BcBIR1*** **transgenic strains following apoptosis-inducing treatments.** (**A**) Microscopic visualization of apoptotic markers following H_2_O_2_ treatment. Fungi were grown for 24 h in PDB medium, H_2_O_2_ was added to a final concentration of 8 mM, cultures were incubated for additional 4 h and then stained and visualized under the microscope. Similar results were obtained following treatment with 250 mM lovastatin and 1.5 mM hexanoic acid. ROS (top) was detected after staining with DHR-123 using the rhodamine filter. Bar  =  100 µm. Chromatin condensation (middle) was detected following nuclei staining with DAPI or Hoechst 3342 using the DAPI filter. Bar  =  5 µm. DNA strand breaks (bottom) were detected following TUNEL assay using the GFP filter. Bar  =  5 µm. (**B-D**) Growth rate and accumulation of apoptotic markers in cultures at stationary phase. (**B**) Fungi were grown for 96 h in 24-well plates and biomass was recorded daily. (**C**) Relative ROS levels. Data represent mean ±SEM of five independent experiments performed in triplicates, (**D**) chromatin condensation and DNA breaks were recorded at 96 h time point, at which stage wild type cultures reach a stationary phase. Data represent mean ±SEM for triplicate samples of 200 nuclei per sample (n = 3). Columns and lines not connected with the same letter are statistically different (p<0.05) according to one-way ANOVA (p<0.001) followed by a post-hoc Tukey HSD analysis.
(TIF)

Figure S4 **Detection of PCD in** ***B. cinerea*** **invasive hyphae using the H1-GFP tagged strains and by measurement of apoptotic markers.** (**A**) Microscopic visualization of nuclei in the H1-GFP tagged strain following H_2_O_2_ treatment. Fungi were grown for 24 h in PDB medium, H_2_O_2_ was added to a final concentration of 10 mM, the cultures were incubated for additional 6 h or 12 h and stained with Hoechst 3342. Images were captured using the GFP and DAPI filter sets. (**B**) Culture of the H1-GFP strain was treated with H_2_O_2_ (as in A), and TUNEL assay was performed on mycelium collected 12 h after H_2_O_2_ treatment, when the H1 nuclear signal was completely disappeared. Note that in this assay the nuclear GFP signal indicates apoptotic nuclei, just the opposite of the H1-GFP marker. (**C**) Beans (*Phaseolus vulgaris)* and peas (*Pisum sativum*) plants were inoculated with spores of the H1-GFP expressing strains. Samples were stained with DAPI or Hoechst 3342 and images were captured using the DAPI and GFP filter sets. (**D**) *A. thaliana* Col-0 wild type plants were inoculated with *B. cinerea* wild type B05.10 strain. Samples were stained with DAPI and images were captured using the DAPI filter set. Although the signal is retained for a longer time compared with the H1-GFP tag (starts to disappear already at 36 h), there is major reduction in the amount of DAPI stained nuclei 48 h PI, and then recovery at 72 h PI. In all images the bar  =  20 µm.
(TIF)

Figure S5 **Nuclear degradation in invasive hyphae of necrotrophic and hemibiotrophic fungi.** Plants were inoculated with spores of H1-GFP (*C. heterostrophus*, *C. gloeosporioides*) or H1-RFP (*M. oryzae*) expressing strains. Infected leaves were photographed at 24, 48 and 72 h PI, except in *C. heterostrophus,* in which infection progresses faster, and therefore images were taken at 6, 12, and 24 h PI. Disappearance of the nuclear GFP signal indicating massive cell death during primary lesion formation and recovery thereafter, is evident in the necrotrophic fungus *C. heterostrophus* (*Ch-Zm*: on corn, at 12 h PI). In the hemibiotrophic fungi *C. gloeosporioides* (*Cg-Pa*: on peas) and *M. oryzae* (*Mo-Os*, on rice) the nuclei are stable and are detectable throughout the infection process. Bar  =  20 µm.
(TIF)

Figure S6 **PCD of** ***B. cinerea*** **wild type and** ***BcBIR1-*** **transgenic strains on** ***A. thaliana*** **Col-0 wild type and** ***pad3*** **mutant plants.** Plants were inoculated with 6 µl droplets of spore suspensions and relative PCD levels were determined by counting the number of condensed and TUNEL stained nuclei. Data represent mean ±SEM for triplicate samples of 200 nuclei per sample (n = 3). The statistical significance of the effect of fungal genotype and infection phase on PCD level was determined using two-way ANOVA. Columns not connected with the same letter are statistically different (p<0.05) according to the two-way ANOVA (p<0.001) followed by a post-hoc Tukey HSD analysis.
(TIF)

Table S1 **Primers used in this study.** *Introduced restriction sites are underlined.
(DOC)
