# Supplementary figures and images for: Anti-Apoptotic Machinery Protects the Necrotrophic Fungus Botrytis cinerea from Host-Induced Apoptotic-Like Cell Death during Plant Infection
Source: PLoS Pathog. 2011 Aug 18;7(8):e1002185. doi: 10.1371/journal.ppat.1002185 (PMC3158046; doi:10.1371/journal.ppat.1002185)

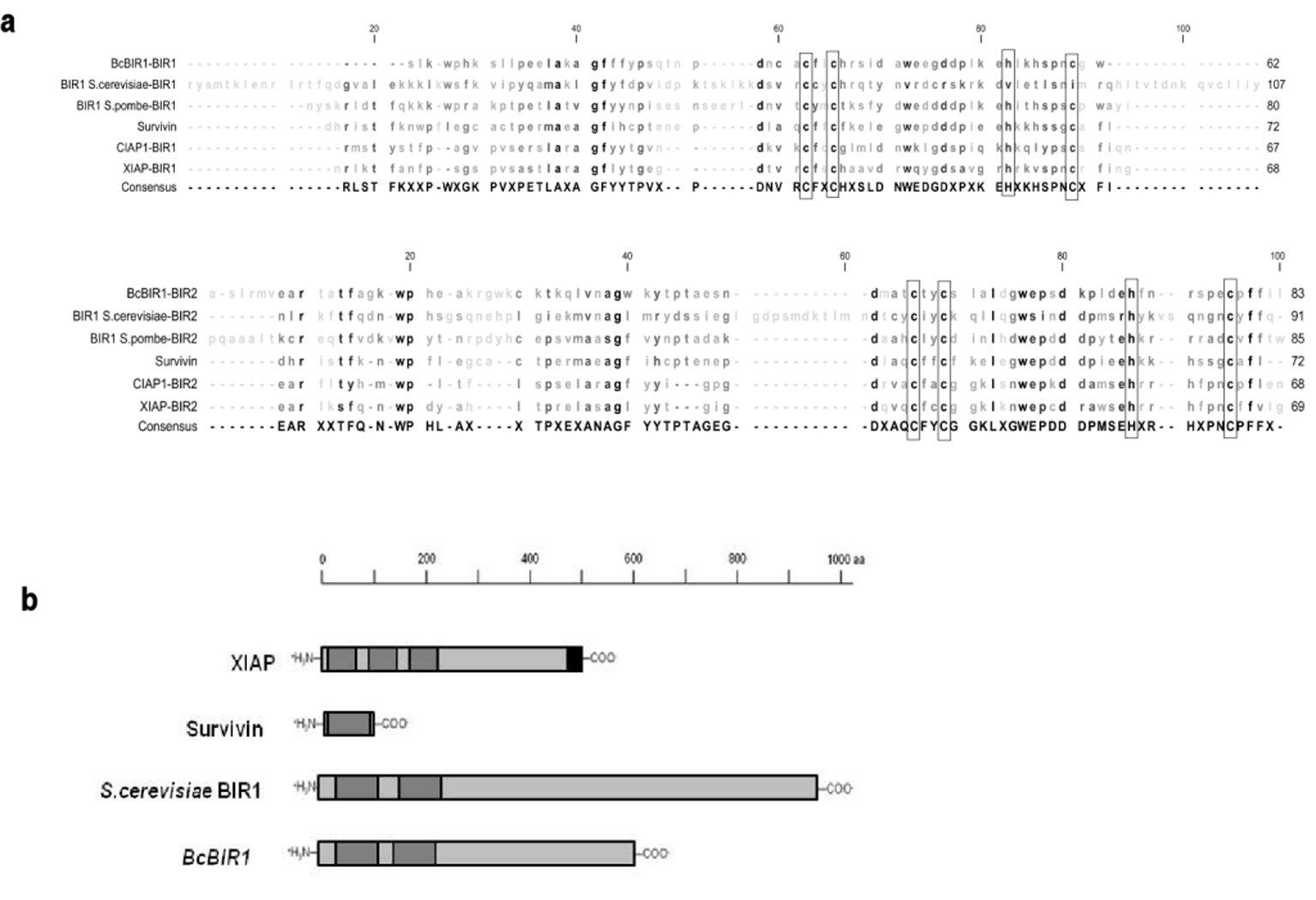

Supplement: Figure S1 — The B. cinerea BcBIR1 is a homologue of BIR1 from S. cerevisiae . (A) Alignment of the amino acid sequence of the two BIR domains in the predicted B. cinerea BcBir1 protein with BIR domains of S. cerevisiae (P47134) and S. pombe (CAA20434) Bir1p, and human XIAP (NP_001158), c-IAP (NP_001157) and survivin (NP_001159). The alignment was generating using ClustalW. Numbers on the top indicate amino acid residue positions. The conserved C2HC residues are marked by black squares. (B) A diagram comparing the organization of BIR domains in human XIAP (Type I IAP) and Survivin (Type II IAP), S. cerevisiae Bir1p and B. cinerea BcBIR1. The BIR domains are colored dark gray. The black square in XIAP denotes a RING domain. (TIF) [file ppat.1002185.s001.tif]

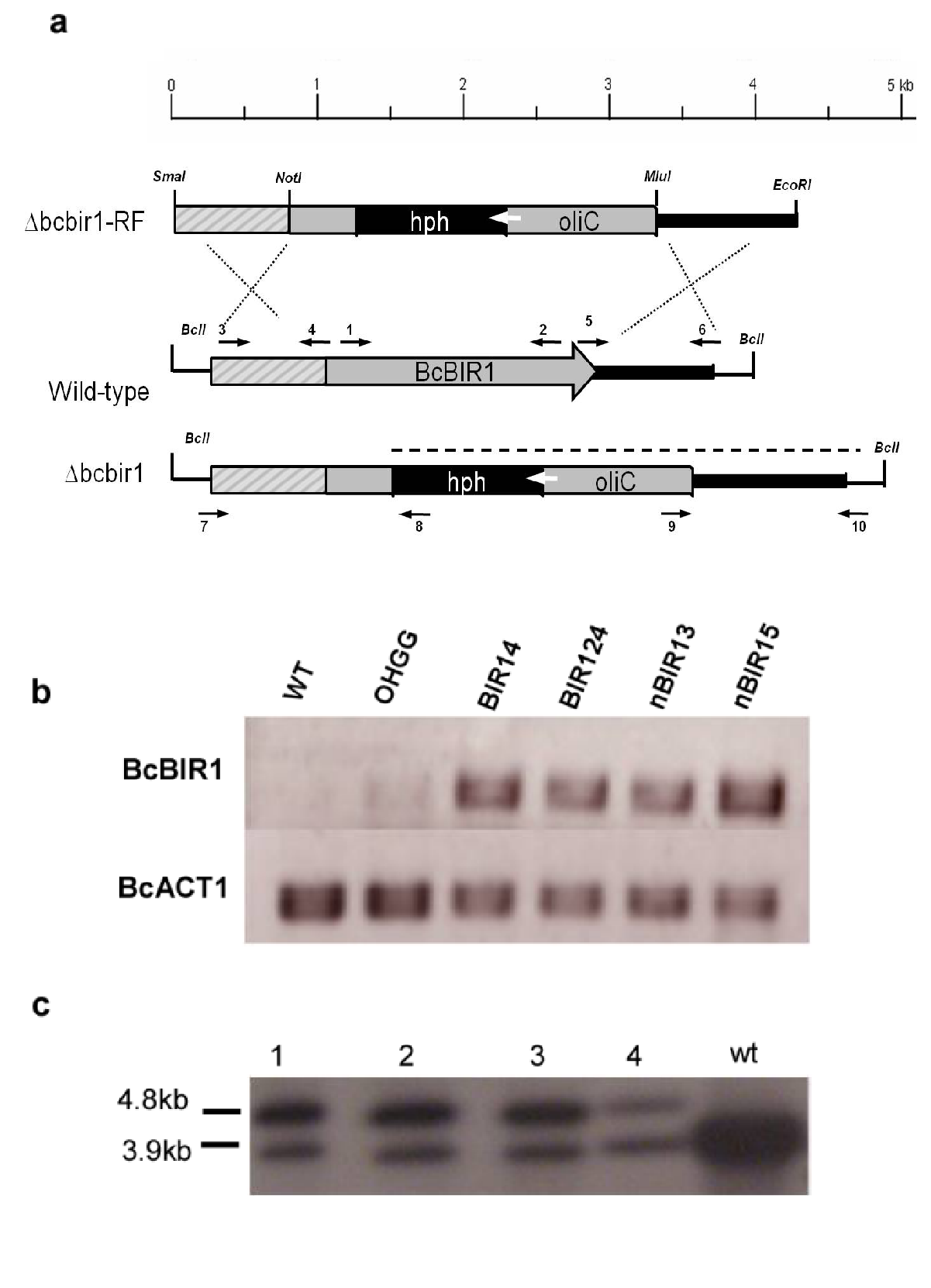

Supplement: Figure S2 — Generation of BcBIR1 over expression and knockout strains. (A) Diagrams of the BcBIR1 knockout vector and genomic locus. Primers for PCR analyses and vector construction are marked by arrows. (B) RT-PCR analysis of hygromycin-resistant colonies obtained from transformation with the BcBIR1 over expression vector pKSHAIG (BIR14, BIR124) and the BcBIR1 N' part over expression vector pKSHANIG (nBIR13, nBIR15). OHGG denotes a transgenic strain expressing free GFP. (C) Southern blot analysis of hygromycin-resistant colonies obtained from transformation with the BcBIR1 replacement vector pΔbcbir1. Genomic DNA from hygromycin-resistant transformants and the wild type strain were digested with BclI. Blots were probed with the fragment that is marked by a dashed line in (A). Wild type nuclei produce a band of 3.9 kb, gene replacement events produce a band of 4.8 kb. (TIF) [file ppat.1002185.s002.tif]

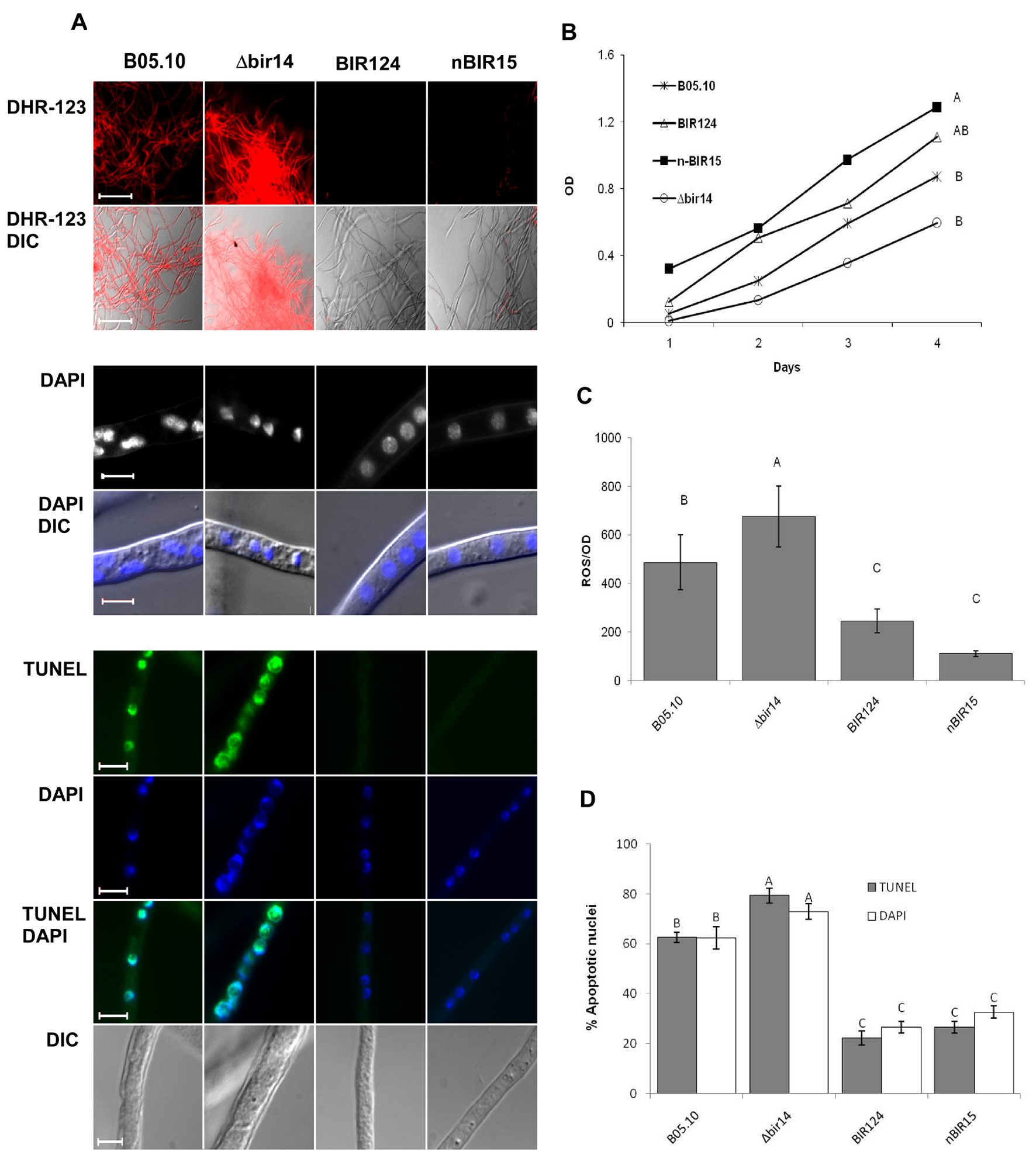

Supplement: Figure S3 — Apoptotic markers in wild type and BcBIR1 transgenic strains following apoptosis-inducing treatments. (A) Microscopic visualization of apoptotic markers following H2O2 treatment. Fungi were grown for 24 h in PDB medium, H2O2 was added to a final concentration of 8 mM, cultures were incubated for additional 4 h and then stained and visualized under the microscope. Similar results were obtained following treatment with 250 mM lovastatin and 1.5 mM hexanoic acid. ROS (top) was detected after staining with DHR-123 using the rhodamine filter. Bar = 100 µm. Chromatin condensation (middle) was detected following nuclei staining with DAPI or Hoechst 3342 using the DAPI filter. Bar = 5 µm. DNA strand breaks (bottom) were detected following TUNEL assay using the GFP filter. Bar = 5 µm. (B-D) Growth rate and accumulation of apoptotic markers in cultures at stationary phase. (B) Fungi were grown for 96 h in 24-well plates and biomass was recorded daily. (C) Relative ROS levels. Data represent mean ±SEM of five independent experiments performed in triplicates, (D) chromatin condensation and DNA breaks were recorded at 96 h time point, at which stage wild type cultures reach a stationary phase. Data represent mean ±SEM for triplicate samples of 200 nuclei per sample (n = 3). Columns and lines not connected with the same letter are statistically different (p<0.05) according to one-way ANOVA (p<0.001) followed by a post-hoc Tukey HSD analysis. (TIF) [file ppat.1002185.s003.tif]

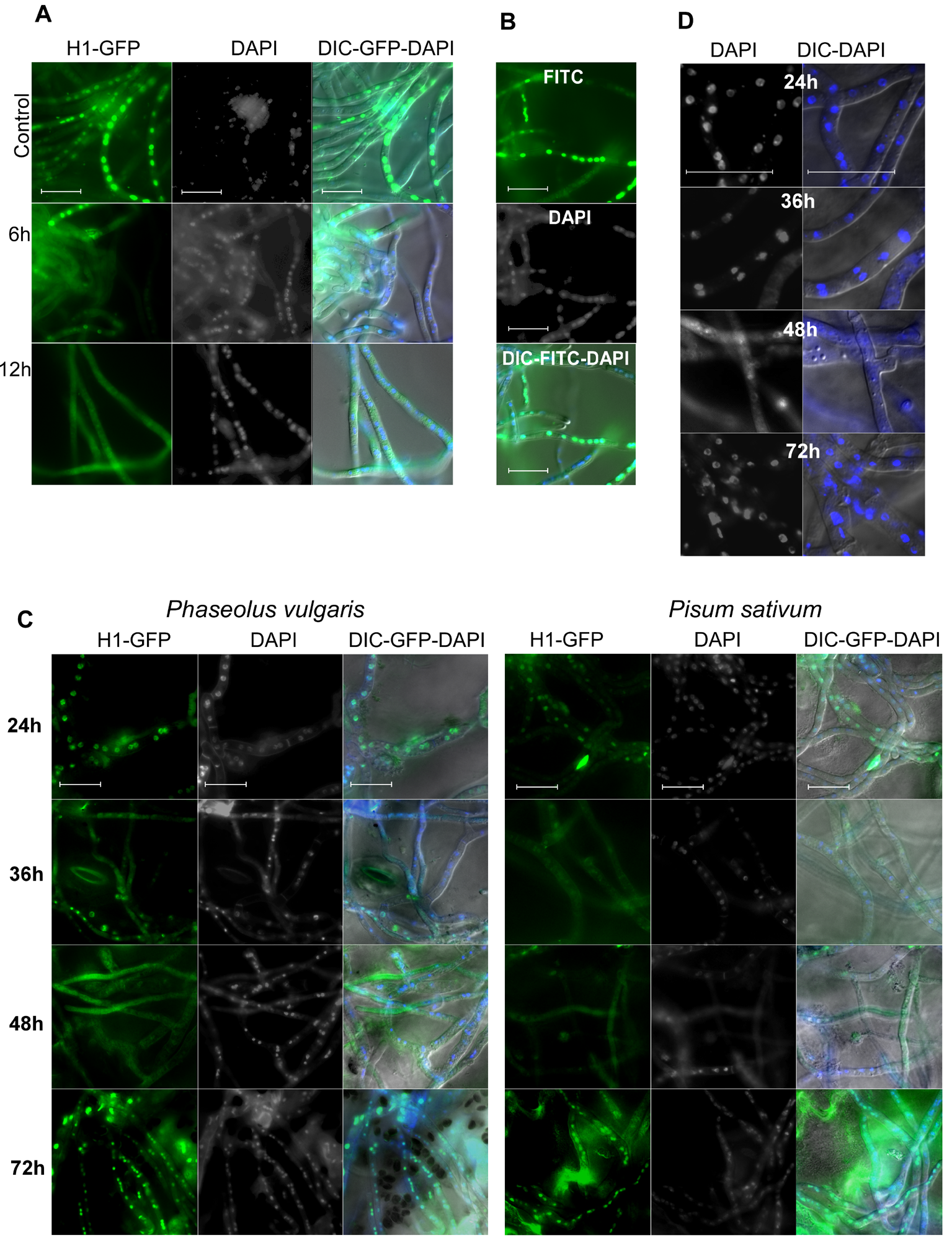

Supplement: Figure S4 — Detection of PCD in B. cinerea invasive hyphae using the H1-GFP tagged strains and by measurement of apoptotic markers. (A) Microscopic visualization of nuclei in the H1-GFP tagged strain following H2O2 treatment. Fungi were grown for 24 h in PDB medium, H2O2 was added to a final concentration of 10 mM, the cultures were incubated for additional 6 h or 12 h and stained with Hoechst 3342. Images were captured using the GFP and DAPI filter sets. (B) Culture of the H1-GFP strain was treated with H2O2 (as in A), and TUNEL assay was performed on mycelium collected 12 h after H2O2 treatment, when the H1 nuclear signal was completely disappeared. Note that in this assay the nuclear GFP signal indicates apoptotic nuclei, just the opposite of the H1-GFP marker. (C) Beans (Phaseolus vulgaris) and peas (Pisum sativum) plants were inoculated with spores of the H1-GFP expressing strains. Samples were stained with DAPI or Hoechst 3342 and images were captured using the DAPI and GFP filter sets. (D) A. thaliana Col-0 wild type plants were inoculated with B. cinerea wild type B05.10 strain. Samples were stained with DAPI and images were captured using the DAPI filter set. Although the signal is retained for a longer time compared with the H1-GFP tag (starts to disappear already at 36 h), there is major reduction in the amount of DAPI stained nuclei 48 h PI, and then recovery at 72 h PI. In all images the bar = 20 µm. (TIF) [file ppat.1002185.s004.tif]

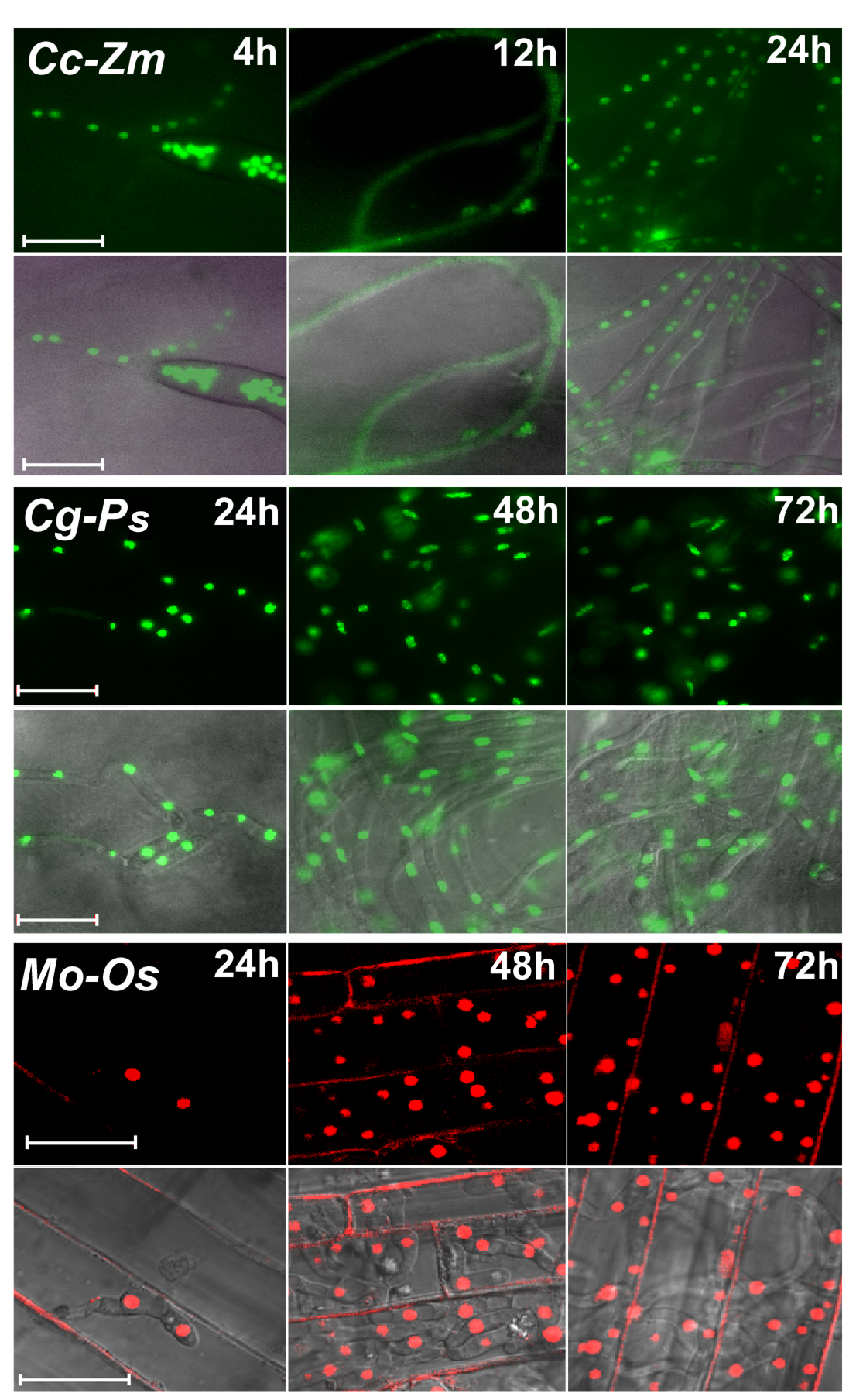

Supplement: Figure S5 — Nuclear degradation in invasive hyphae of necrotrophic and hemibiotrophic fungi. Plants were inoculated with spores of H1-GFP (C. heterostrophus, C. gloeosporioides) or H1-RFP (M. oryzae) expressing strains. Infected leaves were photographed at 24, 48 and 72 h PI, except in C. heterostrophus, in which infection progresses faster, and therefore images were taken at 6, 12, and 24 h PI. Disappearance of the nuclear GFP signal indicating massive cell death during primary lesion formation and recovery thereafter, is evident in the necrotrophic fungus C. heterostrophus (Ch-Zm: on corn, at 12 h PI). In the hemibiotrophic fungi C. gloeosporioides (Cg-Pa: on peas) and M. oryzae (Mo-Os, on rice) the nuclei are stable and are detectable throughout the infection process. Bar = 20 µm. (TIF) [file ppat.1002185.s005.tif]

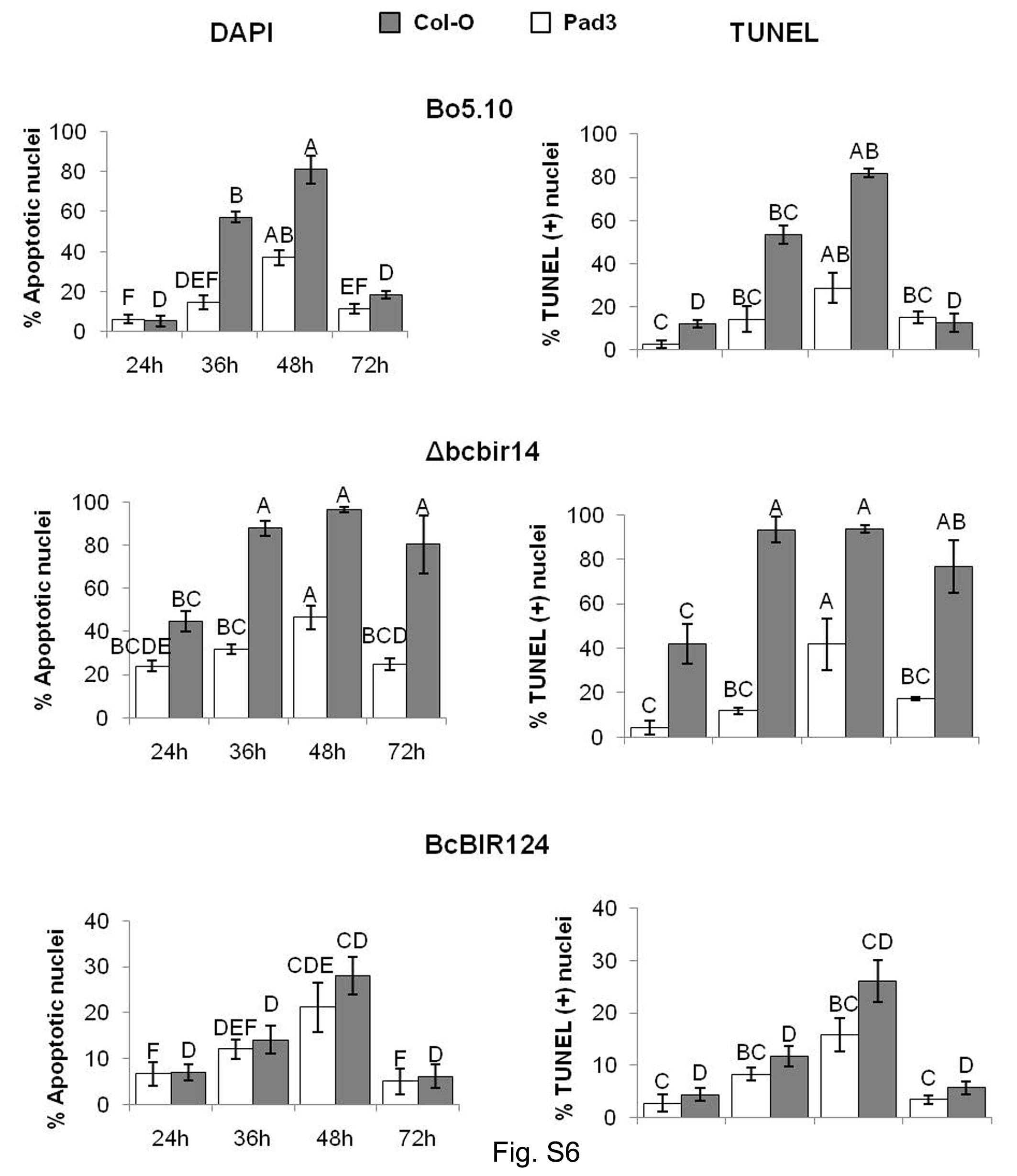

Supplement: Figure S6 — PCD of B. cinerea wild type and BcBIR1- transgenic strains on A. thaliana Col-0 wild type and pad3 mutant plants. Plants were inoculated with 6 µl droplets of spore suspensions and relative PCD levels were determined by counting the number of condensed and TUNEL stained nuclei. Data represent mean ±SEM for triplicate samples of 200 nuclei per sample (n = 3). The statistical significance of the effect of fungal genotype and infection phase on PCD level was determined using two-way ANOVA. Columns not connected with the same letter are statistically different (p<0.05) according to the two-way ANOVA (p<0.001) followed by a post-hoc Tukey HSD analysis. (TIF) [file ppat.1002185.s006.tif]
