# Supplementary material for: Anti-Apoptotic Machinery Protects the Necrotrophic Fungus Botrytis cinerea from Host-Induced Apoptotic-Like Cell Death during Plant Infection
Source: PLoS Pathog. 2011 Aug 18;7(8):e1002185. doi: 10.1371/journal.ppat.1002185 (PMC3158046; doi:10.1371/journal.ppat.1002185)
Supplement: Table S1 — Primers used in this study. *Introduced restriction sites are underlined. (DOC) [file ppat.1002185.s007.doc]

| **#** | **Name** | ***Primer sequence (5'−3')** |
| --- | --- | --- |
| 1 | BIR1F PacI | CCGTTAATTAATGGCTGTTCATGATGTTGC |
| 2 | BIR1R AscI | CCG GGC GCG CCT CAA ACA ATT TCC |
| 3 | BIR1KO5’F SmaI | ATCCCGGG TATCAACTGCGCTCTACAGCCTGG |
| 4 | BIR1KO5’R NotI | ATATGCGGCCGCGTCGACAGACCCTTTTGGAGATTGC |
| 5 | BIR1KO3’F MluI | ATACGCGTCATTTCTTGCAAGCATTGTCGAATC |
| 6 | BIR1KO3’R SacI | ATGAGCTCCAATAAAGCAAATGATCAAAAGG |
| 7 | HR5’F | GACATGCCGTATTATATCAACTGCG |
| 8 | HR5’R | CACAAAATGCAGATGAGACACCG |
| 9 | HR3’F | ATGGGTCCGATAAAATGGTACTGCC |
| 10 | HR3’R | CAAAATCTCTCACGGGCAATAAAGC |
| 11 | *00642*T F AscI | TAGGCGCGCCCGTATGTAGATAAGATGTATGATTAGG |
| 12 | *00642*T R NotI | CGAAGCGGCCGCGGTTGTCAAATCAGATG |
| 13 | N’BIR1 R AscI | TAGGCGCGCCAATACTGTCTCCGTCTTCGAC |
| 14 | *ACT1*P F SpeI | CCGACTAGTTGACTCTCACATCTCG |
| 15 | *ACT1*P R PacI | CCGTTAATTAAGGTTGATAAATTAAGACG |
| 16 | *ACT1*F | ATGGAAGAAGAAGTCGCAGC |
